# Increased activity of lacrimal gland mast cells are associated with corneal epitheliopathy in aged mice

**DOI:** 10.1038/s41514-023-00099-0

**Published:** 2023-02-27

**Authors:** Elsayed Elbasiony, WonKyung J. Cho, Aastha Singh, Sharad K. Mittal, Driss Zoukhri, Sunil K. Chauhan

**Affiliations:** 1 Schepens Eye Research Institute of Mass Eye and Ear, Harvard Medical School, Boston, MA USA; 2 Department of Ophthalmology, Tufts University School of Medicine, Boston, MA USA; 3 Department of Comprehensive Care, Tufts University School of Dental Medicine, Boston, MA USA

**Keywords:** Ageing, Corneal diseases

## Abstract

The lacrimal gland undergoes significant structural and functional deterioration with aging. Marked with increased inflammation and fibrosis, the aged lacrimal gland is unable to perform its protective function. As a result, the ocular surface becomes highly susceptible to various ocular surface pathologies, including corneal epitheliopathy. We and others have previously shown that mast cells mediate tissue inflammation by recruiting other immune cells. However, despite their well-known characteristics of secreting various inflammatory mediators, whether mast cells contribute to the immune cell aggregation and activation, and acinar dystrophy of the aged lacrimal gland has not been investigated. Here, we demonstrate the role of mast cells in age-related lacrimal gland pathophysiology using mast cell-deficient (cKit^w-sh^) mice. Our data demonstrated a significant increase in mast cell frequencies and immune cell infiltration in the lacrimal gland of aged mice. Interestingly, mast cell deficiency resulted in a substantial reduction in inflammation and preservation of lacrimal gland structure, suggesting that mast cells mediate the aging process of the lacrimal gland.

## Introduction

Aging is a process defined by the conglomeration of physiological changes that lead to the deterioration of functional properties at the cellular, tissue, and organ level^1^. Often associated with the dysregulation of the immune system, aging increases the risk of many disorders^2,3^. During aging, lacrimal glands undergo significant structural alterations resulting in ductal obstruction and subsequent acinar atrophy and fibrosis^4^. In conjunction with structural changes, aged lacrimal glands show a marked increase in lymphocytic infiltration^5^. Lacrimal gland, a major component of the lacrimal functional unit, plays a critical role in maintaining tear film homeostasis^1,6^. Accumulation of structural damage and immune dysregulation hampers lacrimal gland function and results in various ocular pathologies, including dry eye disease and corneal epitheliopathy.

Mast cells, tissue-resident immune cells, are distributed throughout most organs in the body, including glandular tissues such as the lacrimal gland^7,8^. Beyond their well-characterized role in allergic immune responses, mast cells are now recognized as multifunctional effector immune cells^9–11^ and have been associated with various pathological conditions, including fibrotic disease^12,13^ and chronic inflammation^14,15^. By releasing preformed and de novo synthesized inflammatory mediators, mast cells contribute to the acute inflammatory response and the progression of chronic diseases^11^. Recently, several studies have shown a significant increase in plasma levels of inflammatory mediators, cytokines, and acute-phase proteins in aged patients^16,17^. Organ senescence, in essence, results from chronic stimulation of both innate and adaptive immune systems^18,19^. Despite the unique characteristics of mast cells in secreting various inflammatory mediators and cytokines, few studies have explored the contribution of mast cells in aging.

In this study, we utilized mast cell-deficient cKit^w-sh^ mice to assess the contribution of mast cells in age-related lacrimal gland deterioration. Our data show that the frequency and activation of mast cells are significantly upregulated in the lacrimal gland of aged mice in conjunction with inflammation and acinar atrophy. However, aged cKit^w-sh^ mice did not display age-related deterioration of glandular acini nor significant immune cell infiltration. In addition, significantly less age-related corneal epitheliopathy was observed in mast cell-deficient mice, suggesting that mast cells orchestrate inflammation-mediated lacrimal gland dystrophy and subsequent functional dysregulation observed during aging.

## Results

### Lacrimal glands of aged mice show increased mast cell frequencies

**Fig. 1:**
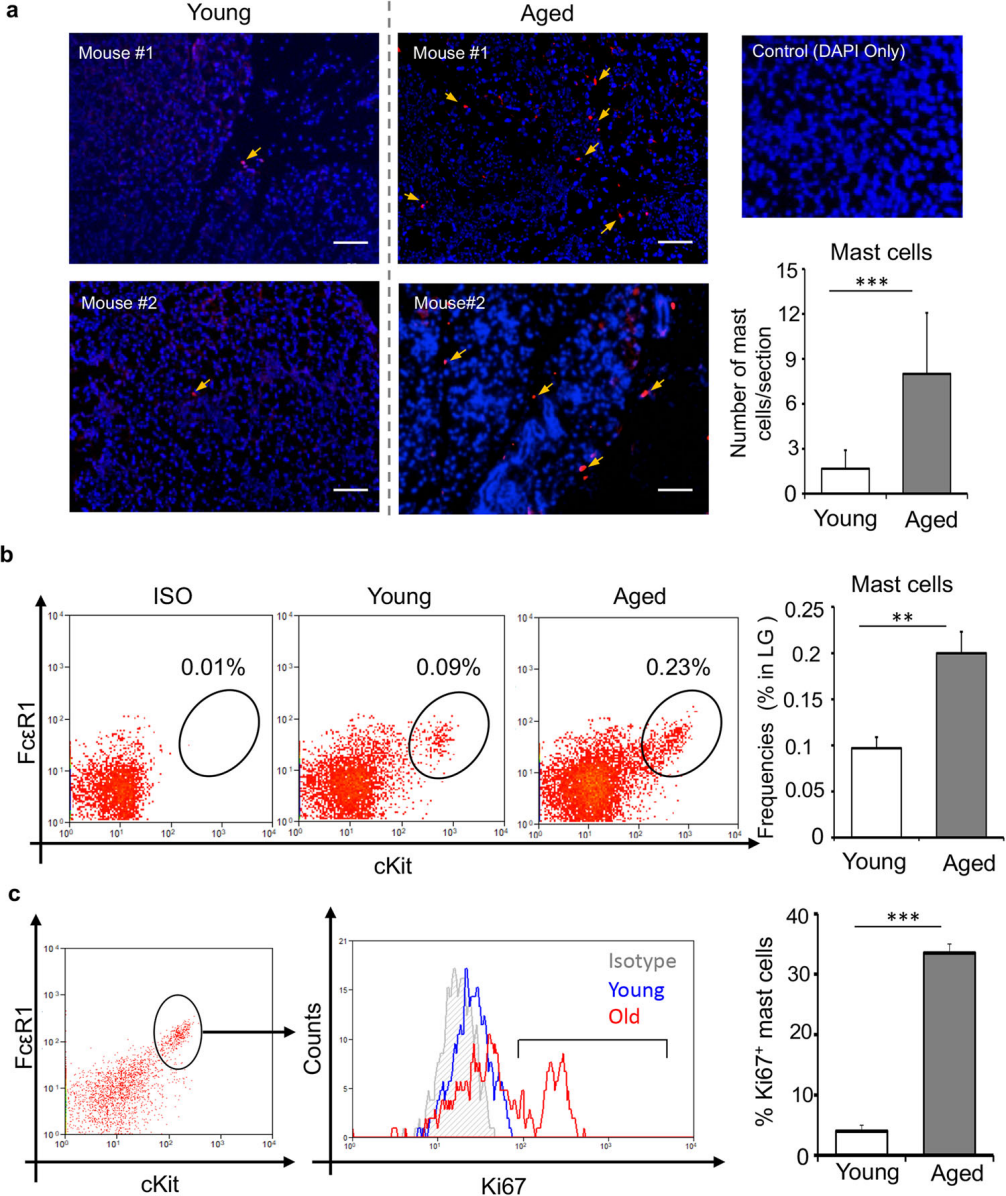
Increased mast cell frequencies in the lacrimal glands of aged mice. **a** Representative immunohistochemistry micrographs (left) and cumulative bar chart (right) showing mast cell distribution per lacrimal gland cross-section (Scale bar, 100 µm). Lacrimal gland of C57BL/6 mice were immunostained with fluorescent-conjugated avidin (Texas Red) showing mast cells (yellow arrows). **b** Representative flow cytometry dot plots (left) and cumulative bar chart (right) showing the frequencies of FcεR1^+^cKit^+^ mast cells, gated on CD45^+^ cells (Gating strategy in Supplementary Fig. S1), in the lacrimal gland of young and aged C57BL/6 mice, compared to isotype-stained control. **c** Representative flow cytometry histogram (left) and cumulative bar chart (right) showing frequencies of Ki67^+^ mast cells in the lacrimal gland of young and aged mice, compared to isotype-stained control. Cumulative data (mean ± SD) from three independent experiments are shown, with each experiment consisting of n of 4 to 6 mice/group. **p < 0.01, ***p < 0.001.

To study the infiltration and distribution of mast cells in the lacrimal gland, young and aged C57BL/6 mice were euthanized to harvest the lacrimal glands. The lacrimal glands were immunostained with mast cell-specific avidin (TexasRed)^20^. Lacrimal glands of young mice showed a fewer number of mast cells; however, a significant increase in mast cell number was observed predominantly in the interlobular space of the lacrimal glands of the aged mice (*p* < 0.001) (Fig. 1a). To further confirm our observation, we studied the frequencies of mast cell infiltration into the lacrimal gland of aged mice using flow cytometry. Single-cell suspensions were prepared from the lacrimal gland of young and aged mice and immunostained with FcεR1 and cKit antibodies. Consistent with our immunohistochemistry data, flow cytometry analysis demonstrated a significant increase in FcεR1^+^cKit^+^ mast cell frequencies in the lacrimal gland of aged mice, relative to that of young mice (*p* < 0.01) (Fig. 1b). Interestingly, a higher percentage of mast cells expressed Ki67 in the lacrimal gland of aged mice, compared to young controls (*p* < 0.001), suggesting that the increase in mast cell frequencies is due to higher proliferation (Fig. 1c). Taken together, our data indicate that frequency of mast cells increases in the lacrimal gland with age and that mast cells are located mainly in the interlobular space.

### Aged mice show increased activation of mast cells at the lacrimal gland and the ocular surface

**Fig. 2:**
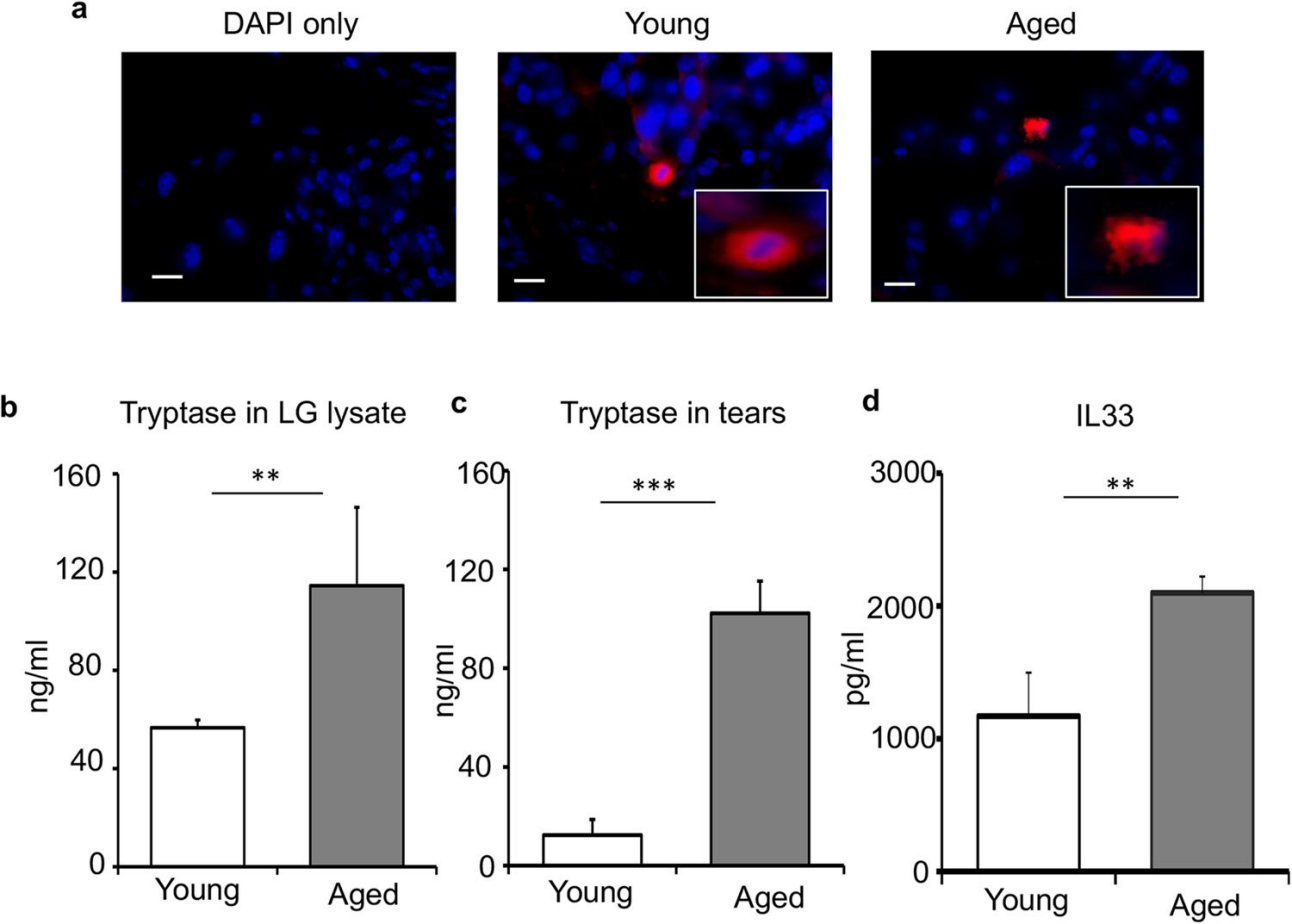
Increased mast cell activation in the lacrimal gland and at the ocular surface of aged mice. **a** Representative immunohistochemistry micrographs of young and aged C57BL/6 mice lacrimal glands stained with fluorescent-conjugated avidin (Texas Red), capturing degranulating mast cell in the lacrimal gland of aged mice (scale bar, 10 µm). **b** Bar chart depicting tryptase levels in the lacrimal gland (LG) lysates of young and aged mice. **c** Bar chart quantifying levels of tryptase in ocular surface tear wash. Ocular surface tear wash of young and aged mice was collected, and mast cell activation was assessed by measuring the levels of tryptase. **d** Bar chart depicting levels of IL33 in the lacrimal gland lysates of young and aged mice. Cumulative data (mean ± SD) from three independent experiments are shown, with each experiment consisting of n of 4 to 6 mice/group. **p < 0.01, ***p < 0.001.

Having observed increased frequencies of mast cells in the lacrimal gland of aged mice, we next sought to determine the effect of aging on mast cell function. Avidin-stained cross-sections of lacrimal glands, harvested from aged mice, showed mast cells undergoing degranulation, as visualized by the release of avidin-stained heparin into the extracellular space (Fig. 2a)^20^. However, this pattern of degranulation was not observed in the lacrimal gland of young mice (Fig. 2a). To further evaluate mast cell activation, total tryptase levels in the lacrimal gland lysates were quantified. Lacrimal glands of aged mice showed a significant 2.5-fold higher level of tryptase as compared to that of young mice (*p* < 0.01) (Fig. 2b). Consistent with increased mast cell activation in the lacrimal gland, we observed a significant 4-fold higher secretion of tryptase in the tear wash collected from the ocular surface of aged mice relative to young mice (*p* < 0.001) (Fig. 2c). Furthermore, we observed an increase in the expression of interleukin 33 (IL33), a potent activator of mast cells^9^, in the lacrimal gland of aged mice, compared to young controls (*p* < 0.01) (Fig. 2d). Taken together, these data suggest that aging results in higher and persistent activation of mast cells.

### Aged mice exhibit inflammatory lacrimal gland and corneal epitheliopathy

**Fig. 3:**
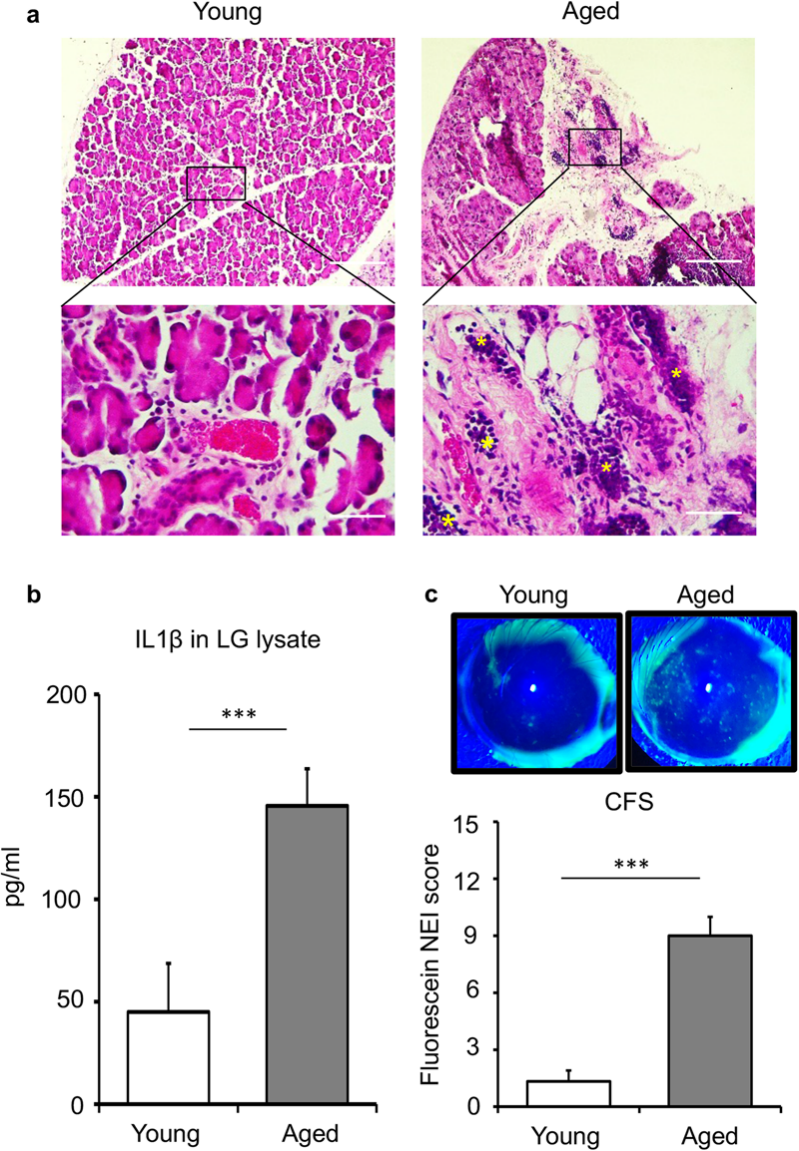
Aged mice showing increased immune cell infiltration in the lacrimal gland and corneal epitheliopathy. **a** Cross-sections of young and aged C57BL/6 mice lacrimal glands were stained with hematoxylin and eosin to visualize immune cell foci (yellow stars) and acinar atrophy (scale bar, 100 µm (upper); 20 µm (lower)). **b** Bar chart depicting protein levels of IL1β in the lacrimal gland lysates of young and aged mice, using ELISA analysis. **c** Representative slit-lamp images (upper panel) and cumulative bar chart (lower panel) measuring corneal fluorescein-staining (CFS) of young and aged mice. Cumulative data (mean ± SD) from three independent experiments are shown, with each experiment consisting of *n* of 4 to 6 mice/group. ***p < 0.001.

Upon mast cell activation, mast cells release a plethora of preformed inflammatory mediators that cause tissue damage^21^. Given our observation of increased activation of mast cells and previous publications showing increased inflammation in aged lacrimal glands^22^, we sought to investigate whether age-mediated changes in the inflammatory milieu result in structural and functional changes. H&E analysis of lacrimal gland cross-sections showed an increase in immune cell foci in aged mice compared to young mice. In addition, substantial tissue fibrosis and acinar atrophy were observed in the lacrimal gland of aged mice (Fig. 3a). Exacerbation of inflammation in aged mice was further confirmed by the elevated levels of the proinflammatory cytokine interleukin 1β (IL1β) in lacrimal gland lysates, as evaluated by ELISA analysis. Indeed, a 3-fold increase in IL1β levels in lysates was observed in the aged lacrimal glands (*p* < 0.001) (Fig. 3b), relative to young controls. These data show that aging exacerbates inflammation in the lacrimal gland and disrupts the glandular structure.

The lacrimal gland is a key organ in maintaining ocular surface homeostasis^1,6^; thus, we sought to investigate the effect of the aged lacrimal gland on the ocular surface. To evaluate corneal epitheliopathy, fluorescein staining was performed, and images were analyzed using a standardized National Eye Institute (NEI) fluorescein staining score^23^. Our data demonstrate a significant increase in the punctated fluorescein staining in the corneas of aged mice relative to that of young mice (*p* < 0.001) (Fig. 3c). These data suggest that the increase in immune foci and acinar atrophy in the lacrimal gland of aged mice is associated with an increase in corneal epitheliopathy.

### Aged mast cell-deficient mice exhibit less lacrimal gland immune cell infiltration

**Fig. 4:**
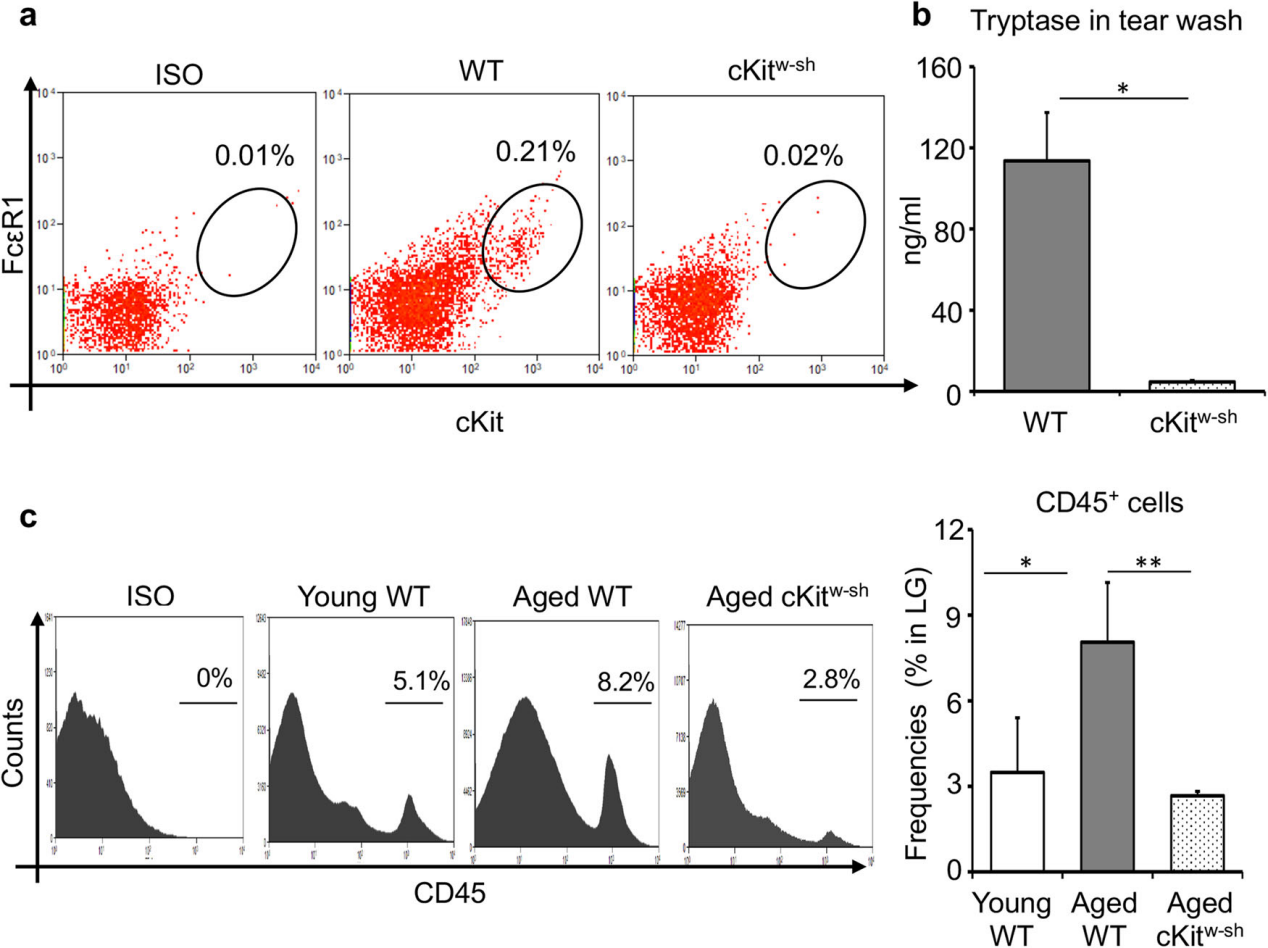
Reduced infiltration of immune cells in the lacrimal gland of aged mast-deficient mice. **a** Representative flow cytometry dot plots confirming the deficiency of FcεR1^+^cKit^+^ mast cells, gated on CD45^+^ cells (Gating strategy in Supplementary Fig. S1), in the lacrimal glands of cKit^w-sh^ mice, compared to wildtype C57BL/6 control (WT). **b** Bar chart depicting tryptase levels in tear wash collected from the ocular surface of WT and cKit^w-sh^ mice. **c** Representative flow cytometry histogram (left) and bar chart depicting frequencies of total CD45^+^ leukocyte in the lacrimal gland of young and aged WT C57BL/6, and aged cKit^w-sh^ mice (Gating strategy in Supplementary Fig. S1). Cumulative data (mean ± SD) from three independent experiments are shown, with each experiment consisting of n of 4 to 6 mice/group. *p < 0.05, **p < 0.01.

Having demonstrated that mast cells increase in frequency and activation in the lacrimal gland of aged mice, we next sought to determine whether mast cells contribute to the age-related high immune cell infiltration of the lacrimal gland by utilizing mast cell-deficient cKit^w-sh^ mice. Our flow cytometry analysis confirmed the deficiency of FcεR1^+^cKit^+^ mast cells in the lacrimal gland of cKit^w-sh^ mice (Fig. 4a). Consistently, negligible levels of tryptase were observed in the tears of cKit^w-sh^ mice, relative to age-matched wild-type C57BL/6 mice (Fig. 4b). To evaluate the total number of leukocytes in the lacrimal gland of aged cKit^w-sh^ mice, single-cell suspensions of the lacrimal gland were stained with anti-CD45 antibody for flow cytometry analysis. A significant increase in the total CD45^+^ immune cell frequencies was observed in the lacrimal gland of aged wild-type mice compared to young wild-type controls (*p* < 0.05). However, this increase was not observed in the lacrimal gland of mast cell-deficient mice, as demonstrated by the significant reduction in the frequencies of CD45^+^ immune cells (Fig. 4c). These data demonstrate that mast cell deficiency results in a significant reduction in the total immune cell infiltration into the lacrimal gland of aged mice.

### Mast cell deficiency prevents age-associated acinar atrophy and corneal epitheliopathy

**Fig. 5:**
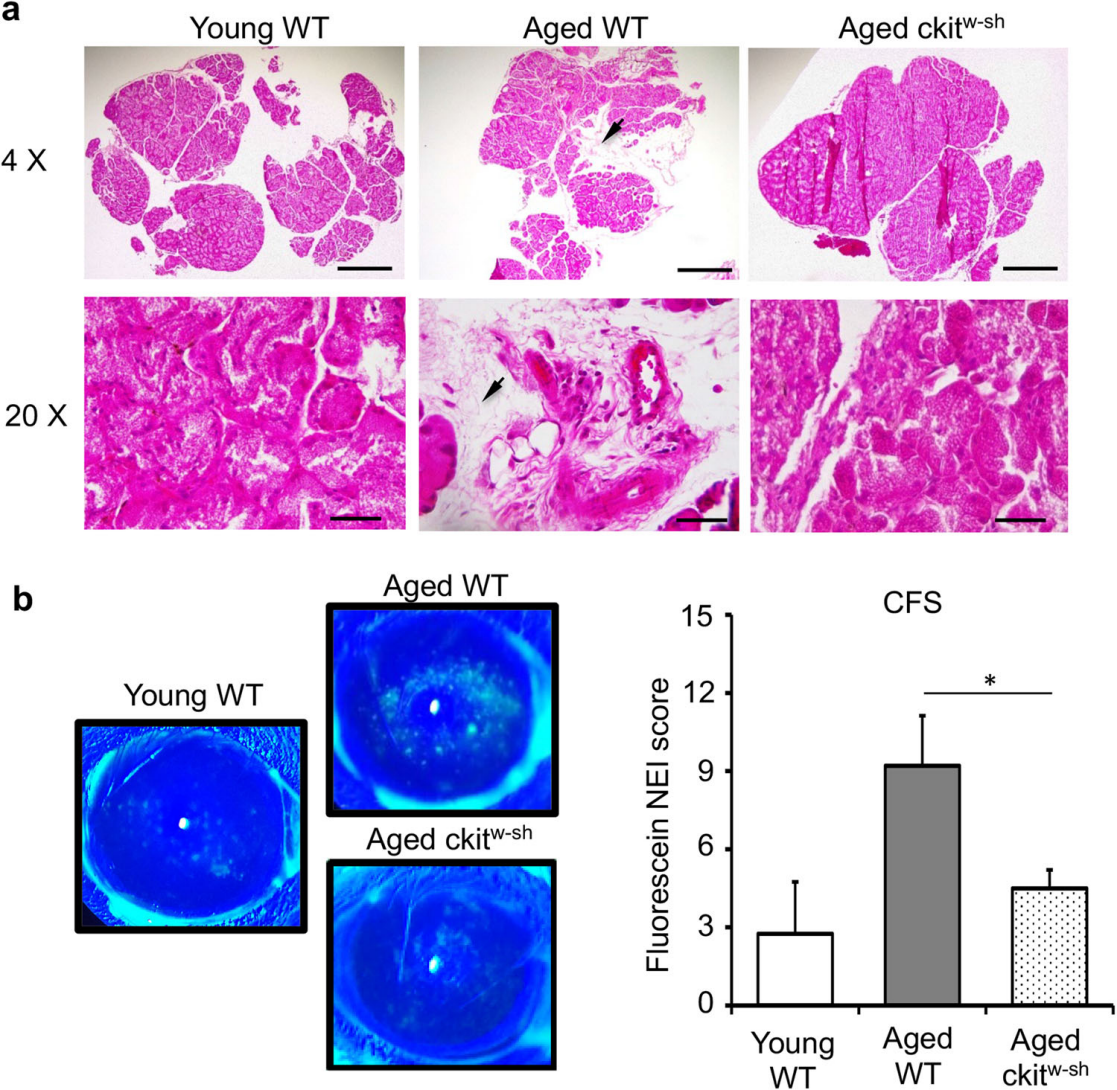
Decreased acinar atrophy and corneal epitheliopathy in aged mast-cell deficient mice. **a** Cross-sections of lacrimal glands of young and aged wildtype C57BL/6 (WT), and aged cKit^W-sh^ mice stained with hematoxylin and eosin to visualize tissue atrophy (black arrow) and immune cell infiltration (scale bar, 500 µm (upper); 50 µm (lower)). **b** Representative slit-lamp images (left) and cumulative bar chart (right) quantifying corneal fluorescein staining (CFS) of the indicated mice. Cumulative data (mean ± SD) from three independent experiments are shown, with each experiment consisting of n of 4 to 6 mice/group. *p < 0.05.

Finally, to confirm the contribution of mast cells in promoting age-related lacrimal gland atrophy and subsequent corneal epitheliopathy, we first evaluated the lacrimal gland tissue structure in mast cell-deficient mice. Unlike the significant loss of tissue architecture and excessive immune cell infiltration observed in the lacrimal gland of aged wildtype mice, lacrimal gland cross-sections of age-matched cKit^w-sh^ mice, stained with H&E, showed preserved gland acini and a lack of immune cells (Fig. 5a). Finally, we investigated whether mast cell deficiency affects age-related corneal epitheliopathy using corneal fluorescein-staining. A significantly smaller area of damaged epithelium was observed in the mast cell-deficient mice, compared to age-matched controls (*p* < 0.05) (Fig. 5b). Collectively, our data demonstrate that mast cell deficiency results in the abrogation of the age-related lacrimal gland atrophy and subsequent corneal epitheliopathy.

## Discussion

Aging is a complex natural process that involves every molecule, cell, and organ in the body^24^. Behavioral changes and exposure to environmental risk factors further contribute to the aging process^25^. The ocular surface is particularly prone to external environmental exposure as it constantly interfaces the environment. In the lacrimal gland, aging results in an increase in tissue inflammation, which subsequently leads to acinar atrophy and fibrosis^1^. Aged lacrimal gland ridden with dilated and tortuous secretory ducts have pronounced ductal obstruction^4,5^. We have previously reported that ocular surface mast cells are the first responders to initiate the onset of acute inflammation^9,26^. In the current study, we observed that the age-related increase in lacrimal gland inflammation is associated with an increase in mast cell frequency. Furthermore, our data show the degranulation of activated mast cells in the lacrimal gland of aged mice, as evidenced by an increase in the tryptase levels in the lacrimal gland and at the ocular surface. Moreover, mast cell deficiency resulted in reduced age-related acinar atrophy and corneal epitheliopathy.

Mast cells, renowned for their role in the allergic immune response, release a plethora of preformed and newly synthesized cytokines and chemokines that recruit a variety of immune cells to the site of allergen exposure^27,28^. Recently, using models of non-allergic inflammation, we and others have reported various ocular pathologies result from non-IgE-mediated activation of mast cells^9,29,30^. As long-lived immune cells, mast cells proliferate in its resident microenvironment and have been shown to increase with aging in the skin^31^. Given the inflammatory function of ocular surface mast cells in mediating tissue damage, our current study investigated the role of mast cells in aged-related pathological deterioration of the lacrimal gland. We observed that mast cells increase in frequency in the lacrimal gland of aged mice and are localized mainly in the interlobular space. Of note, Ki67 nuclear antigen positivity of mast cells in aged lacrimal gland suggests the increased frequencies are primarily due to their active proliferation in the lacrimal gland.

Exposure to environmental stressors have been shown to activate stress-sensing neural pathways to release neuromediators, such as Substance P and calcitonin gene-related peptide (CGRP), which promote ocular inflammation^32–34^. Interestingly, we observed an increase in the expression of IL33, a danger-associated molecular pattern (DAMP)^9^, in the lacrimal gland of aged mice. Recent reports have shown that DAMPs are associated with sterile inflammation caused by aging^35–37^. Our observation of high levels of IL33 in the lacrimal gland and unique barrier function of ocular surface, suggest the external stressors contribute to the high activation of mast cells in aged lacrimal gland.

The lacrimal glands, along with the ocular surface, constitute the lacrimal functional unit to maintain tear film homeostasis and corneal integrity^38,39^. Thus, the dysfunction of the lacrimal gland results in various epitheliopathic disorders, including dry eye disease^38,40^. In the clinic, the population of patients suffering from dry eye disease substantially increases with age, suggesting that the aged lacrimal gland, marked with fibrosis, significantly impairs ocular surface homeostasis^41,42^. Consistent with previous reports^1,22^, we observed various morphological changes in the structure of the lacrimal gland of aged mice, such as periductal fibrosis and acinar atrophy. Interestingly, structural damage observed in the aged lacrimal gland was accompanied by excessive infiltration of immune cells. Given the increase in mast cell activation with aging, our data collectively suggest that mast cells contribute to the inflammation-induced structural damage of aged lacrimal gland.

To confirm the contribution of mast cells in age-related alterations of lacrimal gland structure and the loss of corneal integrity, we utilized aged mast cell-deficient cKit^w-sh^ mice^43^ and studied the morphological alteration and inflammation in the lacrimal gland. Mast cell deficiency resulted in a significant reduction in the age-related infiltration of immune cells into the lacrimal glands^5^. Specifically, we observed a dramatic downregulation of total CD45^+^ immune cell infiltration in the lacrimal gland of aged cKit^w-sh^ mice. Moreover, no age-related acinar atrophy and glandular fibrosis were observed in cKit^w-sh^ mice compared to age-matched wild-type controls. Our experiments using mast cell-deficient mice strongly indicate that mast cells are crucial for immune cell recruitment to the aged lacrimal gland and subsequent acinar atrophy. With the preservation of lacrimal gland structure, mast cell-deficient mice did not display the aged-mediated loss of corneal integrity, as evidenced by the absence of corneal epitheliopathy.

In summary, our findings provide insights into the role of mast cells in age-associated chronic inflammation and tissue dystrophy. Aged lacrimal glands, displaying structural atrophy and fibrosis, exhibit increased mast cell frequencies and activation. Furthermore, abrogation of age-related inflammation in the lacrimal glands of mast cell-deficient mice suggests mast cells are major mediators of chronic inflammation in aged lacrimal glands.

## Methods

### Animals

Six-to 8-week-old (young) and 8-to-12 month-old (aged) C57BL/6 wild-type and age-matched mast cell-deficient cKit^w-sh^ mice, congenic to C57BL/6 mice, (Stock No: 012861) were purchased from the Jackson Laboratory (Bar Harbor, ME) for the described experiments. cKit^w-sh^ mice were confirmed for their deficiency in mast cells at the ocular surface and the lacrimal gland using flow cytometry. cKit^w-sh^ mice, unlike the other mast cell-deficient strain (cKit^w-v^), have a comparable generation of CD45^+^ immune cells in the bone marrow^9,43^. The mice were housed in the Schepens Eye Research Institute animal vivarium. All experiments were reviewed and approved by the Schepens Eye Research Institute Animal Care and Use Committee. The mice were treated according to the ARVO Statement for the Use of Animals in Ophthalmic and Vision Research.

### Lacrimal gland tissue harvesting

Carbon dioxide (CO_2_) inhalation was used to euthanize the mice to harvest the lacrimal glands. The main extraorbital lacrimal gland was accessed through an incision made between the lateral commissure of the eye and ear. By using forceps, the gland was separated from the parotid gland and exteriorized and harvested.

### Lacrimal gland tissue digestion

Single-cell suspensions were prepared from lacrimal glands as previously described^44^. The lacrimal glands were digested in collagenase digestion solution (DMEM media containing 464 U/ml of Collagenase II, 8 U/mL of DNAse I, 2 mM L-glutamine, 1% non-essential amino acids and 1% penicillin/streptomycin) (Lonza, Walkersville, MD, USA) for 50 minutes at 37 °C and 100 RPM. During the digestion, the tissue was broken up by triturating every 15 minutes with increasingly smaller pipette tips. The digested tissue was filtered through a 100 µm mesh and washed two times with 2 mM EDTA in Ca^2+^ and Mg^2+^ -free phosphate-buffered saline (PBS) to stop collagenase digestion. Next, digested tissue was incubated with TrypLE Express (Invitrogen, Carlsbad, CA) for 2 minutes at 37 °C to prepare single cells. Thereafter, TrypLE Express was diluted with 1 U/mL DNAse I in DMEM for 3 min followed by washing with 10% fetal bovine serum (FBS) in DMEM to inactivate any residual trypsin activity.

### Flow cytometry

Single-cell suspensions were stained with fluorochrome-conjugated anti-CD45 (1:100; Cat# 103105), -cKit (1:100; Cat# 105811), -FcεR1 (1:100; Cat# 103105) or -Ki67 (1:100; Cat# 151211) antibodies. Isotypes used were PE Rat IgG2b, κ Isotype Ctrl (Cat# 400607), APC Rat IgG2b, κ Isotype Ctrl (Cat# 400611), Pacific Blue Armenian Hamster IgG Isotype Ctrl (Cat# 400925), and FITC Rat IgG2b, κ Isotype Ctrl (Cat# 400633)^45,46^. Antibodies and respective isotype controls were purchased from BioLegend (San Diego, CA, USA). LSR II flow cytometer (BD Biosciences, San Jose, CA, USA) and Summit software (Dako Colorado, Inc., Fort Collins, CO, USA) were used to acquire and analyze the cells.

### Tryptase assays

Mast Cell Degranulation Assay Kit (Sigma–Aldrich) were used to quantify levels of tryptase^47^. The kit detects the chromophore p-nitroaniline (pNA) cleaved from the labeled substrate tosyl-gly-pro-lys-pNA. In brief, the lacrimal gland lysates and the ocular surface tear wash were incubated with 0.1 mg/mL tosyl-gly-pro-lys-pNA (substrate) for 2 hours at 37 °C. A SpectraMax Plus 384 Microplate Reader (Molecular Devices, San Jose, CA, USA) was used to quantify free pNA at 405 nm.

### Enzyme-linked immunosorbent assay (ELISA)

The lacrimal glands harvested from young and aged mice were lysed in PBS by three complete freeze-thaw cycles (−80 °C for 10 minutes, 37 ^o^C water bath for 5 minutes). Levels of IL1β and IL33 in the lacrimal gland lysates were quantified using commercially available ELISA kits (BioLegend, San Diego, CA, USA), as per the manufacturer’s instructions.

### Immunohistochemistry

The lacrimal glands were harvested and formalin-fixed paraffin-embedded (FFPE) sections (4 µm) were prepared and blocked with 2% BSA and anti-FcR antibodies (catalog #14-0161-86, Affymetrix eBioscience)^48,49^. Sections were stained with avidin-TexasRed (ThermoFisher) overnight at 4 °C. Slides were then mounted using DAPI-containing VECTASHIELD® mounting medium (Vector Laboratories) and examined under a fluorescence microscope (Nikon Eclipse E800; Nikon Instruments, Melville, NY, USA).

### Histology

Cross-sections were prepared from formalin-fixed lacrimal glands harvested from young and aged mice, as previously described^22^. Briefly, the harvested lacrimal gland were fixed in 10% formalin and cross-sectioned in 4 µm thickness. The sections were stained with hematoxylin and eosin to visualize tissue morphology. Corneal tissue structure was analyzed under a brightfield microscope (Nikon Eclipse E800; Nikon Instruments, Melville, NY, USA).

### Slit-lamp microscopy and corneal epitheliopathy scoring

Corneal epitheliopathy was examined by applying 1 μL of 2.5% sodium fluorescein (vital staining) on the ocular surface using a micropipette. After 3 minutes, the ocular surface was visualized using a slit-lamp biomicroscope under cobalt blue light and digital images of corneal epithelial defects were taken. The degree of corneal epitheliopathy was scored according to the National Eye Institute (NEI) grading scale. In brief, the NEI grading scale consists of a grid that divides the corneal area into five sections, each of which is assigned a score between 0 and 3 depending on the amount and distribution of the corneal fluorescein staining (CFS); the total CFS score ranges from 0/15 (absence of corneal epitheliopathy) to 15/15 (severe epitheliopathy).

### Statistical analysis

Unpaired two-tailed Student *t*-test was used to compare means between two groups. The significance level was set at *p* < 0.05. Data are presented as means ± standard deviations of three independent experiments. Samples sizes were estimated on the basis of previous experimental studies using mast cell deficient cKit^w-sh^ mice and sterile inflammation^9,45,47^.

## Supplementary information

Supplementary Figure 1

## Data Availability

Included in article: The data that support the findings of this study are available in the methods of this article. Detailed data that support the findings of this study are available from the corresponding author upon reasonable request.
