# Supplementary material for: Increased activity of lacrimal gland mast cells are associated with corneal epitheliopathy in aged mice
Source: NPJ Aging. 2023 Feb 27;9(1):2. doi: 10.1038/s41514-023-00099-0 (PMC9971332; doi:10.1038/s41514-023-00099-0)
Supplement: Supplementary file 1 — Supplementary Figure 1 [file 41514_2023_99_MOESM1_ESM.pdf]

Supplementary Figure 1.

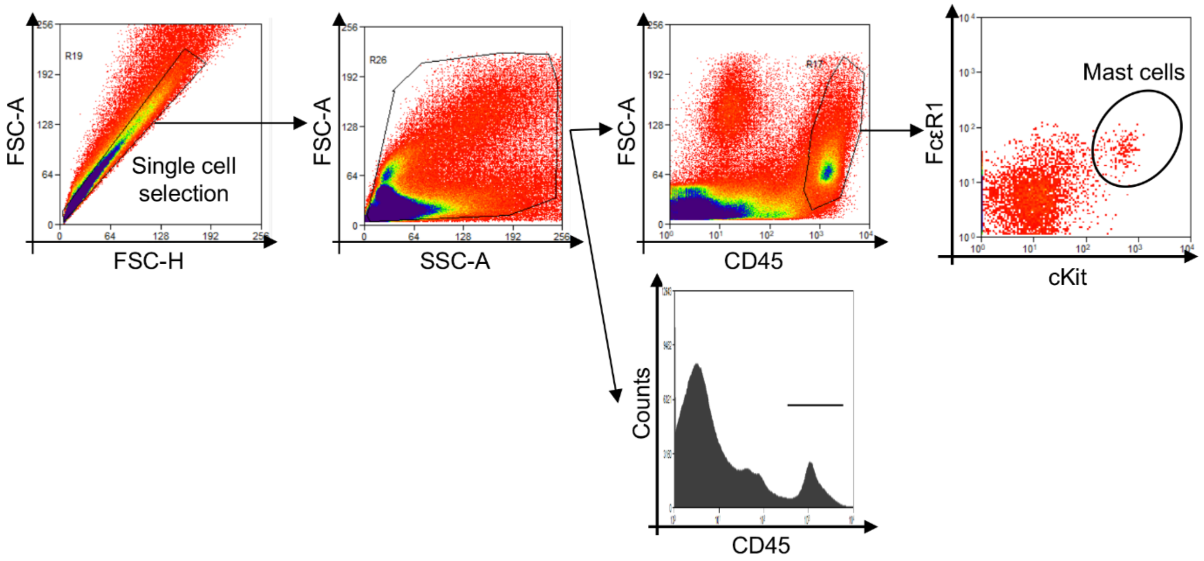

Gating strategies for CD45$^+$ immune cells and FcεR1$^+$cKit$^+$ mast cells. Representative dot plots demonstrating the gating strategy for frequencies of CD45$^+$ immune cells (Fig. 4c) and FcεR1$^+$cKit$^+$ mast cells (Fig. 1b, 1c & 4a) in single cell suspensions of lacrimal glands.
